# Rational modification of an HIV-1 gp120 results in enhanced neutralization breadth when used as a DNA prime

**DOI:** 10.1186/1742-4690-9-S2-P343

**Published:** 2012-09-13

**Authors:** A Wallace, MJ Duenas-Decamp, M Vaine, PJ Peters, S Wang, PR Clapham, S Lu

**Affiliations:** 1 University of Massachusetts Medical School, Worcester, MA, USA

## Background

The identification of phenotypic features of the HIV-1 envelope glycoprotein that correlate with neutralization breadth is an important goal of HIV vaccine research. Recently we compared the immunogenic potential of two gp120s differing in their ability to utilize CD4; B33 (highly macrophage topic) and LN40 (non-macrophage tropic). Using a DNA prime protein boost regimen in New Zealand White Rabbits, LN40-primed sera displayed enhanced breadth compared to the B33-primed group, with differences in immunogenicity between groups modulated by specific residues within and flanking the V3 loop and the CD4bs. To better understand the role of these residues in eliciting breadth, we introduced reciprocal mutations between LN40 and B33 at these critical positions.

## Methods

Three groups of four rabbits were primed with one of three chimeric LN40/B33 gp120 DNAs, followed by a polyvalent protein boost. Time course and endpoint titers were determined via ELISA. Neutralization breadth was analyzed by Monogram against a panel of sixteen viruses using a Phenosense neutralization assay. Anti-gp120 serum specificities were determined using a set of overlapping peptides spanning the entire gp120 via ELISA.

## Results

We found that sera primed with a B33 chimera containing specific LN40 residues within the V3 loop and the CD4 binding loop displayed enhanced neutralization breadth against a cross-clade panel of Tier 1 and 2 viruses compared to the B33-primed group. Interestingly, a second B33 chimera containing two additional LN40 substitutions (Stu-Bsu R373/N386) within C3/V4 primed the broadest response, being broader than even the LN40-primed group. Additionally, peptide ELISAs showed differences in reactivity between priming groups which were most pronounced for the C3/V4 region, suggesting an important role for these regions in modulating serum antibody responses against gp120.

## Conclusion

While the role of R373/N386 is still under investigation, these results represent potentially promising steps towards the rational, targeted design of a better gp120 immunogen.

